# Selective catalytic dehydration of furfuryl alcohol to 2, 2′-difurfuryl ether using a polyoxometalate catalyst

**DOI:** 10.1038/s41598-017-13472-3

**Published:** 2017-10-11

**Authors:** Shaoxiang Yang, Yanfeng Hao, Jialin Wang, Hao Wang, Yimeng Zheng, Hongyu Tian, Yongguo Liu, Baoguo Sun

**Affiliations:** Beijing Advanced Innovation Center for Food Nutrition and Human Health, Beijing Key laboratory of Flavor Chemistry, Beijing Technology and Business University, No.11 Fucheng Road, Haidian District, Beijing, 100048 P. R. China

## Abstract

The spice flavour compound 2, 2′-difurfuryl ether (DFE) is widely utilised in the food industry as it has a coffee-like, nutty, earthy, mushroom-like odour. However, despite intensive research efforts, to date, an environmentally friendly and practical synthetic preparation technique for 2, 2′-difurfuryl ether is still unavailable. Here, we investigate a new approach using polyoxometalate catalysts to selectively catalytically dehydrate furfuryl alcohol to 2, 2′-difurfuryl ether. We have successfully applied this methodology using the polyoxometalate (POMs) catalyst {[(CH_3_CH_2_CH_2_CH_2_)_4_N]_2_[SMo_12_O_40_]} to produce 2,2′-difurfuryl ether in a 30.86% isolated yield.

## Introduction

Furfuryl alcohol (FA) is considered as an important template chemical for the production a range of useful chemicals, such as levulinic acid^1^, alkyl levulinate^2^ and various other useful polymer products^3,4^. FA is synthesised by a selective hydrogenation process from furfural and its conversion into oligomer (Oligomerized FA, OFA) and polymer (Polymerized FA, PFA) products has been widely explored owing to their utility in a range of applications^5^. Several molecular structures of OFAs and PFAs have been proposed using a combination of NMR^6^, IR^7^, UV–Vis^8^, Raman spectroscopy^9^, XRD^10^ and DFT calculations^11^. From these studies a variety of dimer products have been proposed including: 2,2′-difurfuryl ether (DFE); 2,2′-difurylmethane (DFM); 2,2′-difuryl-ethylene (DFEt) and a hydroxyl-carbon bridge dimer^12,13^. Further examples include, 4-furfuryl-2-pentenoic acid γ-lactone (PAL) which can be produced over γ-alumina during FA polymerisation and 2-hydroxymethyl-5(5-furfuryl) furan (HFF) which is a themaleic anhydride product^14^. However, studies reveal that HFF and PAL cannot co-exist in either acid-polymerized or γ-alumina-polymerized FA, although analytical results were not enough to support PAL existence^14^.

A particularly valuable chemical product of FA is 2,2′-difurfuryl ether (DFE)^15^, which is a spice flavour compound with an aroma described as a mixture of coffee and mushroom scents combined with nutty and earthy odours^16,17^. It can be eaten according to the Flavour Extract Manufacturers′ Association (FEMA), Joint FAO/WHO Expert Committee on Food Additives (JECFA) and National Health and Family Planning Commission of PRC (NHFPC) regulation guidelines. DFE is referred to by the FEMA No. 3337, the JECFA No. 1522 and the Chinese Standards for Food Additives No. S1108.

**Figure 1:**
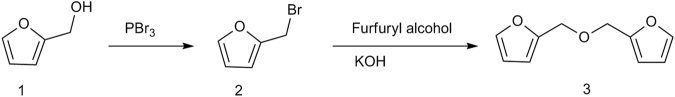
The two-step synthesis of DFE from FA via bromination and etherification reactions.

DFE is synthesised from FA in a two-step process comprised of bromination followed by etherification (Fig. 1)^18^. However, this particular synthetic method poses significant environmental hazards, such as pollution, and thus, a search for an alternative cleaner, safer and more environmentally friendly approach is a key priority^19,20^. Interestingly, DFE can also be obtained as a side-product during FA oligomerization reactions over heterogeneous catalysts^21^. Polyoxometalate (POMs) catalysts are one example of a heterogeneous catalyst which could be used for this purpose; however, to the best of our knowledge, very little quantitative analysis information is available on the presence of DFE during such FA oligomerization reactions. Indeed, we have previously reported, the successful synthesis of another flavour compound (−)-Ambrox, which was prepared using (−)-sclareol as a starting material which was oxidised using hydrogen peroxide in the presence of the POMs catalyst {[C_5_H_5_NC_16_H_33_][H_2_PMo_12_O_40_]}, which is a quaternary ammonium phosphomolybdate catalyst^22^. Therefore, in this study, we investigate the feasibility of using selective catalytic dehydration of furfuryl alcohol in the presence of various POM catalysts to produce 2, 2′-difurfuryl ether - thus producing a more environmentally friendly synthetic approach.

## Results and Discussion

With respect to FA oligomerization reactions, the catalyst tungsten oxide in the liquid phase (100 °C) has been successfully employed to produce a range of OFAs. These include: five dimers (2,2′-difurylmethane, 2-(2-furylmethyl)-5-methylfuran, difurfuryl ether, 4-furfuryl-2-pentenoic acid γ-lactone and 5-fufuryl-furfuryl alcohol) and two trimers (2,5-difurfurylfuran and 2,2′-(furylmethylene)-*bis*(5-methylfuran)) were observed, difurfuryl ether and 5-Furfuryl-furfuryl alcohol were the dominant products^23–25^. Another class of catalysts are POMs, which are discrete metal-oxide clusters containing W, Mo, V or Nb that have attracted increasing interest owing to their multi-electronic redox activities, and photochemical, acidic and magnetic properties. Importantly, there are a wide range of potential applications that POMs can be envisaged for, such as catalysts and functional materials^26^.

As with all catalysis, the first step in utilising POMs for the selective catalytic dehydration of furfuryl alcohol to 2, 2′-difurfuryl ether, will be to choose an appropriate POM catalyst. For thus, a series of POMs catalysts were prepared as summarised in Table 1 ^27–31^. In order to relatively assess the utility of these synthetic catalysts a set of standard experimental conditions was employed (i.e., in toluene at 100 °C for 7 h). The results are given in Table 2, revealing catalytic activities in the following order: sulfo-polyoxometalates > quaternary ammonium phosphomolybdates > quaternary ammonium phosphotungstates and heteropolyacid salts. With respect to the heteropolyacid salts, the catalysts 4 d and 4 h showed greater yields (entry 4, 8 Table 2) than the other heteropolyacid salt catalysts (entry 1–8 Table 2). We also found that the heteropolyacid Al^3+^ salts showed a much better catalytic ability than the Na^+^, K^+^ and Fe^3+^ salts. Furthermore, of the quaternary ammonium phosphomolybdates with the same phosphomolybdic group, we found that the character of the quaternary ammonium cation groups have a very limited influence on the catalytic activity (entries 13–17, Table 2). Moreover, although Mo and W belong to the same main group, they display difference catalytic activities in this reaction. We also found that the quaternary ammonium phosphomolybdates usually displayed better catalytic ability (entries 13–17, Table 2) than the quaternary ammonium phosphotungstates (entries 9–12, Table 2). Overall, of all the POMs tested, the sulfo-polyoxometalate catalyst 4r ({[(CH_3_CH_2_CH_2_CH_2_)_4_N]_2_ [SMo_12_O_40_]}) gave the best yield (26.90%; entry 18, Table 2), and the product was readily isolated and purified.

**Table 1:** Synthesis of the catalysts.

Entry	Catalyst 4	Chemical compositions of catalyst	Yield (%)	IR (cm^−1^)
1	4a	Na_3_PW_12_O_40_	81	1079,976,895, 802
2	4b	FePW_12_O_40_	85	1063,968,897, 807
3	4c	K_3_PW_12_O_40_	79	1079,976,895, 802
4	4d	AlPW_12_O_40_	82	1076,981,897, 803
5	4e	Na_3_PMo_12_O_40_	80	1063,964,893, 802
6	4 f	FePMo_12_O_40_	86	1067,961,893, 802
7	4 g	K_3_PMo_12_O_40_	77	1092,964,893, 802
8	4 h	AlPMo_12_O_40_	80	1064,961,869, 782
9	4i	{[(CH_3_) _4_N][H_2_PW_12_O_40_]}	88	2922,1851,1635,1486,1079,976,895,802
10	4j	{[(CH_3_) _3_C_16_H_33_N][H_2_PW_12_O_40_]}	80	2922,2851,1623,1481, 1062,959,879,803
11	4k	{[C_5_H_5_NC_16_H_33_][H_2_PW_12_O_40_]}	76	2922,2851,1635,1486, 1079,976,895,802
12	4 l	{[(CH_3_CH_2_ CH_2_ CH_2_)_4_N][H_2_PW_12_O_40_]}	83	2971,2867,1615,1474, 1080,976,894, 816
13	4 m	{[(CH_3_) _4_N][H_2_P Mo_12_O_40_]}	88	2922,2851,1635,1471, 1062,956,880,798
14	4n	{[(CH_3_) _3_C_16_H_33_N][H_2_P Mo_12_O_40_]}	72	2922,2851,1671,1471, 1080,977,897, 805
15	4o	{[C_5_H_5_NC_16_H_33_][H_2_PMo_12_O_40_]}	82	2922,2851,1635,1486, 1079,976,895, 802
16	4p	{[(CH_3_CH_2_ CH_2_ CH_2_)_4_N][H_2_P Mo_12_O_40_]}	70	2922,2851,1671,1471, 1080,977,897, 805
17	4q	{[C_5_H_5_NC_16_H_33_]_2_[HPMo_12_O_40_]}	78	2921,2851,1640,1478, 1062, 961, 879, 794
18	4r	{[(CH_3_CH_2_CH_2_CH_2_)_4_N]_2_[SMo_12_O_40_]}	75	2921,2851,1634,1488, 1079, 976, 895, 799

**Table 2:** Optimization of the catalyst.

Entry	Catalyst	DFE yield (%)	Entry	Catalyst	DFE yield (%)
1	4a	2.08	10	4j	7.62
2	4b	5.41	11	4k	6.46
3	4c	7.85	12	4 l	1.41
4	4d	13.30	13	4 m	17.92
5	4e	3.55	14	4n	16.46
6	4 f	3.97	15	4o	16.73
7	4 g	4.90	16	4p	14.06
8	4 h	14.58	17	4q	14.20
9	4i	13.09	18	4r	26.90

^*^Reaction conditions: FA (10 mmol), catalyst (0.1 mmol), toluene (10 mL), 100 °C and 7 h. GC yield.

Whilst POMs were known as effective catalysts, reports generally focus on their chemical oxidation, electrochemical oxidation, reduction reactions, photochemical oxidation, base catalysed reactions, acid catalysis and other reaction potential^32^. In this study, the reasons these different POMs catalysts showed different activities on this selective catalytic dehydration reaction were unclear.

In order to optimise the synthetic conditions for DFE using the 4r POMs catalyst, we systematically varied the parameters of catalyst quantity and reaction time. The amount of catalyst 4r in the reaction was optimised firstly (entries 1–10, Table 3). We found that DFE was produced in the highest yield (26.90%) when 1% equivalent of the catalyst was used (entry 6, Table 3). The yield decreased significantly, from 26.90% to 8.29%, when the catalyst loading was lowered from 1% to 0.1% equivalents, whereas the yield did not increase with incremental catalyst loading from 1% to 5% equivalents. We subsequently optimised the reaction time, the results were shown in Table 3 (entries 11–20). We found that the DFE yield increased gradually with extended reaction times from 1 h to 9 h (entries 11–19, Table 3), however, the yield did not increase furthermore up to 10 h (entries 19, 20, Table 3). Overall, the optimised conditions for DFE synthesis are a reation time of 9 h at 100 °C with a 1% equivalent of 4r catalyst, resulting in a yield of 34.50% (entries 19, Table 3). The reaction was repeated under the above optimised conditions and 2,2′-difurfuryl ether (DFE) was obtained in an average isolated yield of 30.86%^16,17^.

**Table 3:** Optimization of the Reaction Conditions using the 4r catalyst.

Entry	Catalyst amount (mmol)*	Yield (%)	Entry	Reaction time **	Yield (%)
1	0.01	8.29	11	1 h	10.58
2	0.03	14.16	12	2 h	10.99
3	0.05	15.72	13	3 h	15.51
4	0.07	20.65	14	4 h	16.28
5	0.09	23.16	15	5 h	18.47
6	0.1	26.90	16	6 h	19.40
7	0.2	26.12	17	7 h	26.90
8	0.3	26.08	18	8 h	27.24
9	0.4	25.24	19	9 h	34.50
10	0.5	25.07	20	10 h	34.25

^*^Reaction conditions: FA (10 mmol), catalyst 4r (relative equiv.), toluene (10 mL), 100 °C and 7 h. GC yield. **Reaction conditions: FA (10 mmol), catalyst 4r (0.01 equiv.), toluene (10 mL), 100 °C and 10 h. GC yield.

**Figure 2:**
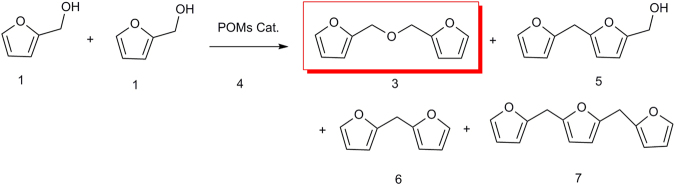
The selective catalytic dehydration process converting furfuryl alcohol to 2,2′-difurfuryl ether using a polyoxometalate (POM) catalyst.

As per previous literature preparations of DFE^25^, other compounds appear in the oligomerization reaction (Fig. 2, Figure S1), as determined by GC/MS. As shown in Table 4, these include: compound 5 (5–furfuryl–furfuryl alcohol, Figure S5); compound 6 (2, 2′–difurylmethane, Figure S6) and compound 7 (2, 5–difurfurylfuran, Figure S7). Although other compounds have been proposed as side-products in such reactions, we found no evidence of them under our experimental and equipment conditions.

**Table 4:** The yields for the oligomerization reaction using the 4r catalyst.

Entry	Reaction time	Compound 5 yield (%)	Compound 6 yield (%)	Compound 7 yield (%)	FA conversion (%)
1	1 h	5.85	6.84	1.63	28.42
2	2 h	6.61	6.33	2.81	30.70
3	3 h	13.97	7.20	5.47	43.27
4	4 h	14.18	11.72	6.53	50.59
5	5 h	18.7	17.03	6.75	57.4
6	6 h	20.2	23.20	10.1	75.1
7	7 h	20.3	20.60	10.86	80.9
8	8 h	17.47	13.80	8.94	82.56
9	9 h	14.20	8.94	6.93	89.81
10	10 h	13.10	6.87	6.81	89.82

^*^Reaction conditions: FA (10 mmol), catalyst 4r (0.01 equiv.), toluene (10 mL) and 100 °C. GC yield.

As shown in Tables 3 and 4, the reaction time has an obvious influence on the yields of compound 4 (DFE), compound 5, compound 6 and compound 7. As expected, the yields of compound 5, compound 6 and compound 7 decrease and yields of compound 4 increases with reaction time. The yield of compound 5 increased gradually with extended reaction times from 1 h to 7 h (entries 1–7, Table 4), but decreased with reaction time from 7 h to 10 h (entries 7–10, Table 4). Compound 5 was obtained in the highest yield of 20.30% after 7 h (entries 7, Table 4). The yield of compound 6 increased gradually with extended reaction times from 1 h to 6 h (entries 1–6, Table 4), but the yield decreased with reaction time from 6 h to 10 h (entries 6–10, Table 4). Compound 6 has the highest yield of 23.20% after 6 h (entries 6, Table 4). The yield of compound 7 increased gradually with extended reaction times from 1 h to 7 h (entries 1–7, Table 4), but the yield decreased with the increment of reaction time from 7 h to 10 h (entries 7–10, Table 4). Compound 7 has highest yield of 10.86% after 7 h (entries 7, Table 4). Therefore, it was fortunate that compound 4 (DFE) was obtained in the highest yield of 34.50% after 9 h (entries 19, Table 3). These results clearly illustrate that catalyst 4r was a strong candidate as a heterogeneous catalyst for the selective catalytic dehydration of FA to DFE.

## Conclusions

In this paper, a comprehensive study on the utility of POMs catalysts for the selective catalytic dehydration of furfuryl alcohol to 2, 2′-difurfuryl ether has successfully been carried out. Through assessing a range of potential POMs catalysts, we found that {[(CH_3_CH_2_CH_2_CH_2_)_4_N]_2_[SMo_12_O_40_]} was the most effective, accomplishing the reaction in an overall 30.86% yield. Thus, we have present a novel synthetic avenue for the efficient and environmentally benign synthesis of 2, 2′-difurfuryl ether, which employs a inexpensive and simple POMs catalyst. Further studies are underway to further improve the yield of 2, 2′-difurfuryl ether using other POMs catalysts and various synthetic conditions.

## Methods

### Synthesis of the catalysts a-h

All of the catalysts a-h were synthesised by the same approach. This method is illustrated following for catalyst 4a as an example.

A solution of H_3_PW_12_O_40_ (2.88 g, 1 mmol) in deionized water (10 mL) was added into a 50 mL beaker. The reaction mixture was stir for 5 min at 25 °C, and Na_2_CO_3_ (1.06 g, 10 mmol) in deionized water (10 mL) was added over 5 min. After addition, the mixture was stir for 1 h at 25 °C, then filtered and washed with deionized water and dried *in vacuo* and subsequently calcined at 450 °C for 2 h to afford 4a as a white solid (2.38 g, 81%)^33^. The elemental analysis data for the purified salts were as follows.

Calculated for 4a Na_3_PW_12_O_40_: Na, 2.34; P, 1.05; W, 74.88%. Found: Na, 2.37; P, 1.11; W, 74.79%.

Calculated for 4b FePW_12_O_40_: Fe, 1.90; P, 1.06; W, 75.22%. Found: Fe, 1.88; P, 1.09; W, 75.29%.

Calculated for 4c K_3_PW_12_O_40_: K, 3.92; P, 1.03; W, 73.68%. Found: K, 3.95; P, 1.07; W, 73.69%.

Calculated for 4d AlPW_12_O_40_: Al, 0.93; P, 1.07; W, 75.97%. Found: Al, 0.90; P, 1.07; W, 76.01%.

Calculated for 4e Na_3_PMo_12_O_40_: Na, 3.65; P, 1.64; Mo, 60.88%. Found: Na, 3.61; P, 1.69; Mo, 60.81%.

Calculated for 4 f FePMo_12_O_40_: Fe, 2.97; P, 1.65; Mo, 61.30%. Found: Fe, 2.93; P, 1.60; Mo, 61.41%.

Calculated for 4 g K_3_PMo_12_O_40_: K, 6.05; P, 1.60; Mo, 59.36%. Found: K, 6.11; P, 1.58; Mo, 59.43%.

Calculated for 4 h AlPMo_12_O_40_: Al, 1.46; P, 1.67; Mo, 62.26%. Found: Al, 1.51; P, 1.69; Mo, 62.20%.

### Synthesis of the catalysts 4i-q

Synthesis of catalysts was illustrated by the synthesis of catalyst 4n.

H_3_P Mo_12_O_40_ (1.82 g, 1 mmol) and deionized water (10 mL) were combined in a 50 mL three-neck flask. The mixture was stirred for 5 min at 25 °C and further cetylpyridinium chloride (0.36 g, 1 mmol) in deionized water (10 mL) was added after 5 min, then the mixture was stirred for 3 h at 25 °C. When filtered, the filtrate cake was washed with liquid and dried by vacuum to produce 4n (1.76 g, 82%) as a dark green solid. The elemental analysis data for the purified salts were as follows.

Calculated for 4i {[(CH_3_)_4_N][H_2_PW_12_O_40_]}: C, 1.63; H, 0.48; N, 0.47; P, 1.05; W, 74.70%. Found: C, 1.59; H, 0.47; N, 0.50; P, 1.09; W, 74.73%.

Calculated for 4j {[(CH_3_)_3_C_16_H_33_N][H_2_PW_12_O_40_]}: C, 7.21; H, 1.40; N, 0.44; P, 0.98; W, 69.73%. Found: C, 7.20; H, 1.43; N, 0.41; P, 1.02; W, 69.71%.

Calculated for 4k {[C_5_H_5_NC_16_H_33_][H_2_PW_12_O_40_]}: C, 7.92; H, 1.27; N, 0.44; P, 0.97; W, 69.30%. Found: C, 7.94; H, 1.29; N, 0.43; P, 0.96; W, 69.34%.

Calculated for 4 l {[(CH_3_CH_2_CH_2_CH_2_)_4_N][H_2_PW_12_O_40_]}: C, 6.16; H, 1.23; N, 0.45; P, 0.99; W, 70.67%. Found: C, 6.16; H, 1.25; N, 0.44; P, 0.98; W, 70.63%.

Calculated for 4 m {[(CH_3_)_4_N][H_2_PMo_12_O_40_]}: C, 2.53; H, 0.74; N, 0.74; P, 1.63; Mo, 60.65%. Found: C, 2.51; H, 0.77; N, 0.75; P, 1.62; Mo, 60.69%.

Calculated for 4n {[(CH_3_)_3_C_16_H_33_N][H_2_PMo_12_O_40_]}: C, 10.82; H, 2.10; N, 0.66; P, 1.47; Mo, 54.59%. Found: C, 10.78; H, 2.07; N, 0.64; P, 1.50; Mo, 54.55%.

Calculated for 4o {[C_5_H_5_NC_16_H_33_][H_2_PMo_12_O_40_]}: C, 11.85; H, 1.89; N, 0.66; P, 1.46; Mo, 54.08%. Found: C, 11.81; H, 1.92; N, 0.65; P, 1.44; Mo, 54.12%.

Calculated for 4p {[(CH_3_CH_2_CH_2_CH_2_)_4_N][H_2_PMo_12_O_40_]}: C, 9.30; H, 1.85; N, 0.68; P, 1.50; Mo, 55.71%. Found: C, 9.34; H, 1.83; N, 0.69; P, 1.53; Mo, 55.69%.

Calculated for 4q {[C_5_H_5_NC_16_H_33_]_2_[HPMo_12_O_40_]}: C, 20.74; H, 3.19; N, 1.15; P, 1.27; Mo, 47.33%. Found: C, 20.70; H, 3.16; N, 1.17; P, 1.26; Mo, 47.37%.

### Synthesis of the catalyst 4r

A solution of Na_2_MoO_4_·2H_2_O (6.05 g, 25 mmol) in deionized water (200 mL) was added into a 500 mL beaker. The reaction mixture was stir for 5 min at 25 °C, and then NH_4_VO_3_ (0.6 g, 5.1 mmol) in H_2_SO_4_ (50 mL, 2 mol/L) was added. The reaction mixture was stir for 5 min, then CH_3_COCH_3_ (250 mL) was added. After stirring for 1 h at 25 °C, tetrabutylammonium bromide (10 g, 31 mmol) was added. After addition, the mixture was stir for 0.5 h at 25 °C, then filtered, washed with deionized water, ethanol and acetonitrile, and dried *in vacuo* to afford 4r as a yellow solid (3.60 g, 75%)^29^. The elemental analysis data for the purified salts were as follows. Calculated for 4r {[(CH_3_CH_2_CH_2_CH_2_)_4_N]_2_[SMo_12_O_40_]}: C, 16.65; H, 3.14; N, 1.21; S, 1.39; Mo, 49.88%. Found: C, 16.66; H, 3.12; N, 1.23; S, 1.42; Mo, 49.87%.

### Synthesis of the DFE

**Figure 3:**
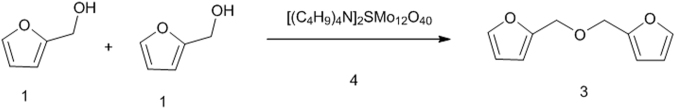
The synthesis of 2,2′-difurfuryl ether using catalyst 4r [(C_4_H_9_)_4_N]_2_SMo_12_O_40_.

Each of the catalysts were employed, respectively, for this reaction and the overall synthetic conditions are illustrated following using 4r as an example (Fig. 3).

FA (0.98 g, 10 mmol), 4r (0.23 g, 0.1 mmol, 1% equiv.) and toluene (10 mL) were added into a 50 mL three-neck flask. The mixture was stirred for 9 h at 100 °C. The toluene was subsequently removed under reduced pressure. The residue was extracted with ether, the organic phases were then washed with a saturated solution of Na_2_CO_3_ and brine and then dried over MgSO_4_. After solvent removal, the residue was purified by flash chromatography on silica gel (petroleum/EtOAc, 40:1) to afford DFE as a colourless liquid (0.55 g, 30.86%).

^1^H NMR (300 MHz, CDCl_3_) δ: 4.48 (4 H, s, -CH_2_-O), 6.34(4 H, s, -CH = CH-), 7.42(2 H, d, J = 0.9 Hz, C = CH-O) (Figure S2).

^13^C NMR (75 MHz, CDCl_3_) δ: 63.38, 109.54, 110.19, 142.81, 151.30 (Figure S3).

MS (ESI), *m/z*: 178.1 [M] ^+^, 147.0, 119.0, 91.1, 53.1 (Figure S4).

## Electronic supplementary material

Supplementary information

## References

[1] Girisuta B, Janssen LPBM, Heeres HJ (2006). A kinetic study on the decomposition of 5-hydroxymethylfurfural into levulinic acid. Green Chem..

[2] Kim T (2014). Thermodynamics and reaction pathways of furfuryl alcohol oligomer formation. Catal. Commun..

[3] Guigo N, Mija A, Vincent L, Sbirrazzuoli N (2007). Chemorheological analysis and model-free kinetics of acid catalysed furfuryl alcohol polymerization. Phys. Chem. Chem. Phys..

[4] Yi B (2006). Catalytic polymerization and facile grafting of poly (furfuryl alcohol) to single-wall carbon nanotube: preparation of nanocomposite carbon. J. Am. Chem. Soc..

[5] Wang H, Zhang L, Gavalas GR (2000). Preparation of supported carbon membranes from furfuryl alcohol by vapor deposition polymerization. J. Membrane Sci..

[6] Choura M, Belgacem NM, Gandini A (1996). Acid-catalyzed polycondensation of furfuryl alcohol: Mechanisms of chromophore formation and cross-linking. Macromolecules.

[7] Batista PS, De Souza MF (2000). Furfuryl alcohol conjugated oligomer pellicle formation. Polymer.

[8] Méalares C, Hui Z, Gandini A (1996). Conjugated polymers bearing furan rings: 1. Synthesis and characterization of oligo (2, 5-furylene vinylene) and its thiophene homologue. Polymer.

[9] Almeida Filho CD, Zarbin AJ (2006). Porous carbon obtained by the pyrolysis of TiO2/poly (furfuryl alcohol) nanocomposite: preparation, characterization and utilization for adsorption of reactive dyes from aqueous solution. J. Brazil. Chem. Soc..

[10] dos Santos Batista P, de Souza MF (1999). Furfuryl alcohol polymerisation inside capillaries. Synthetic met..

[11] Kim T (2011). Acid-Catalyzed Furfuryl Alcohol Polymerization: Characterizations of Molecular Structure and Thermodynamic Properties. ChemCatChem.

[12] Bertarione S (2008). Furfuryl Alcohol Polymerization in H-Y Confined Spaces: Reaction Mechanism and Structure of Carbocationic Intermediates. J. Phys. Chem. B.

[13] Mendonça CR, Batista PS, de Souza MF, Zilio SC (2001). Chemical dynamics and reverse saturable absorption in di-furfuryl ether solutions. Chem. Phys. Lett..

[14] Wewerka EM, Loughran ED, Walters KL (1971). A study of the low molecular weight components of furfuryl alcohol polymers. J. Appl. Polym. Sci..

[15] Khusnutdinov RI (2007). Furfuryl alcohol in synthesis of levulinic acid esters and difurylmethane with Fe and Rh complexes. Russ. J. Appl. Chem..

[16] Bressanello D (2017). Coffee aroma: Chemometric comparison of the chemical information provided by three different samplings combined with GC–MS to describe the sensory properties in cup. Food chem..

[17] Yang N (2016). Determination of volatile marker compounds of common coffee roast defects. Food chem..

[18] Fillion, E. The development of new oxabicyclic-based strategies for the stereo- and enantioselective synthesis of azepines Thiepines and Thiocines, Polysubstituted Decalins and Related Fused Polycycles, Chemistry, Vol. PhD, University of Toronto, 50 (Toronto 1998).

[19] Grondal C, Jeanty M, Enders D (2010). Organocatalytic cascade reactions as a new tool in total synthesis. Nat. chem..

[20] Climent MJ, Corma A, Iborra S (2010). Heterogeneous catalysts for the one-pot synthesis of chemicals and fine chemicals. Chem. Rev..

[21] Meng Q, Zheng H, Zhu Y, Li Y (2016). Study on the reaction pathway in decarbonylation of biomass-derived 5-hydroxymethylfurfural over Pd-based catalyst. J. Mol. Catal. A-Chem..

[22] Yang SX (2016). One-pot synthesis of (−)-Ambrox. Sci. Rep..

[23] Chan X, Nan W, Mahajan D, Kim T (2015). Comprehensive investigation of the biomass derived furfuryl alcohol oligomer formation over tungsten oxide catalysts. Catal. Commun..

[24] Chan X (2016). Catalysts Loading Effect of Tungsten Oxide Catalytic Furfuryl Alcohol Oligomerization. Mater. Today Proceed..

[25] Kim T, Jeong J, Rahman M, Zhu E, Mahajan D (2014). Characterizations of furfuryl alcohol oligomer/polymerization catalyzed by homogeneous and heterogeneous acid catalysts. Korean J. Chem. Eng..

[26] Cronin L, Müller A (2012). Special thematic issue on polyoxometalates. Chem. Soc. Rev..

[27] Zuwei X, Ning Z, Yu S, Kunlan L (2001). Reaction-controlled phase-transfer catalysis for propylene epoxidation to propylene oxide. Science.

[28] Himeno S, Miyashita K, Saito A, Hori T (1990). Preparation of Tetrabutylammonium Dodecamolybdosulfate (VI), [(C_4_H_9_)_4_N]_2_SMo_12_O_40_. Chem. Lett..

[29] Langanke J, Greiner L, Leitner W (2013). Substrate dependent synergetic and antagonistic interaction of ammonium halide and polyoxometalate catalysts in the synthesis of cyclic carbonates from oleochemical epoxides and CO_2_. Green Chem..

[30] Maksimchuk NV (2008). Heterogeneous selective oxidation catalysts based on coordination polymer MIL-101 and transition metal-substituted polyoxometalates. J. Catal..

[31] Rüther T (2003). Electrochemical investigation of photooxidation processes promoted by sulfo-polyoxometalates: coupling of photochemical and electrochemical processes into an effective catalytic cycle. J. Am. Chem. Soc..

[32] Wang SS, Yang GY (2015). Recent advances in polyoxometalate-catalyzed reactions. Chem. Rev..

[33] Chen C (2006). Photodegradation of dye pollutants catalyzed by porous K_3_PW_12_O_40_ under visible irradiation. Environ. Sci. technol..

