# Supplementary material for: Selective catalytic dehydration of furfuryl alcohol to 2, 2′-difurfuryl ether using a polyoxometalate catalyst
Source: Sci Rep. 2017 Oct 11;7:12954. doi: 10.1038/s41598-017-13472-3 (PMC5636835; doi:10.1038/s41598-017-13472-3)
Supplement: Supplementary file 1 — Supplementary information [file 41598_2017_13472_MOESM1_ESM.doc]

**Selective catalytic dehydration of furfuryl alcohol to 2, 2′-difurfuryl ether using a polyoxometalate catalyst**

**Shaoxiang Yang^1^*, Yanfeng Hao^1^, Jialin Wang^1^, Hao Wang^1^, Yimeng Zheng^1^, Hongyu Tian^1^*, Yongguo Liu^1^, and Baoguo Sun^1^**

**^1^** *Beijing Advanced Innovation Center for Food Nutrition and Human Health, Beijing Key laboratory of Flavor Chemistry, Beijing Technology and Business University, No.11 Fucheng Road, Haidian District, Beijing 100048, P.R. China*

^*^ Telephone: +86-10-68985382. Fax: 86-10-68985382. E-mail:

[yangshaoxiang@th.btbu.edu.cn](mailto:yangshaoxiang@th.btbu.edu.cn) (Shaoxiang Yang); [tianhy@btbu.edu.cn](mailto:tianhy@btbu.edu.cn) (Hongyu Tian)

| **TABLE OF CONTENTS** | **PAGE** |
| --- | --- |
| **S1. GC Analysis of the products** | **2** |
| **S2. ^1^H NMR Spectra of 2, 2′-difurfuryl ether** | **3** |
| **S3. ^13^C NMR Spectra of 2, 2′-difurfuryl ether** | **3** |
| **S4. MS Spectra of 2, 2′-difurfuryl ether** | **4** |
| **S5. MS Spectra of compound 5** | **4** |
| **S6. MS Spectra of compound 6** | **5** |
| **S7. MS Spectra of compound 7** | **5** |

**S1. GC Analysis** **of the products**

An Agilent 7890B GC System 5977A MSD GC with a flame ionization detector (FID) was used for GC analyses (Agilent Technologies, Santa Clara, CA, USA). The column used HP-5MS (30.0m×250μm，0.25μm). The analytical condition was as follows: injector temperature 250°C, detector temperature 230°C, He as carrier gas, constant flow mode 1.2 mL/min, split ratio 20/1. The oven temperature was programmed from 50°C to 150°C at a rate of 20°C/min, then to 280°C at a rate of 10°C/min, and held at 240°C for 10 min. The concentration of samples was about 0.5 wt.% in dry ether, the injection volume was about 0.4 μl.


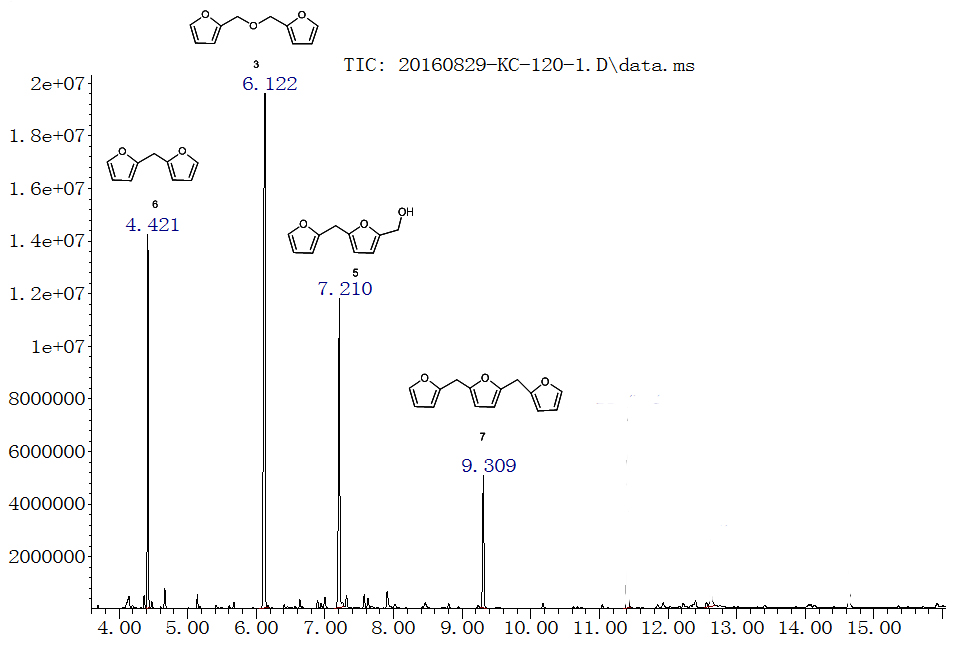


**GC Spectra**

**S2. ^1^H NMR Spectra of 2, 2′-difurfuryl ether**


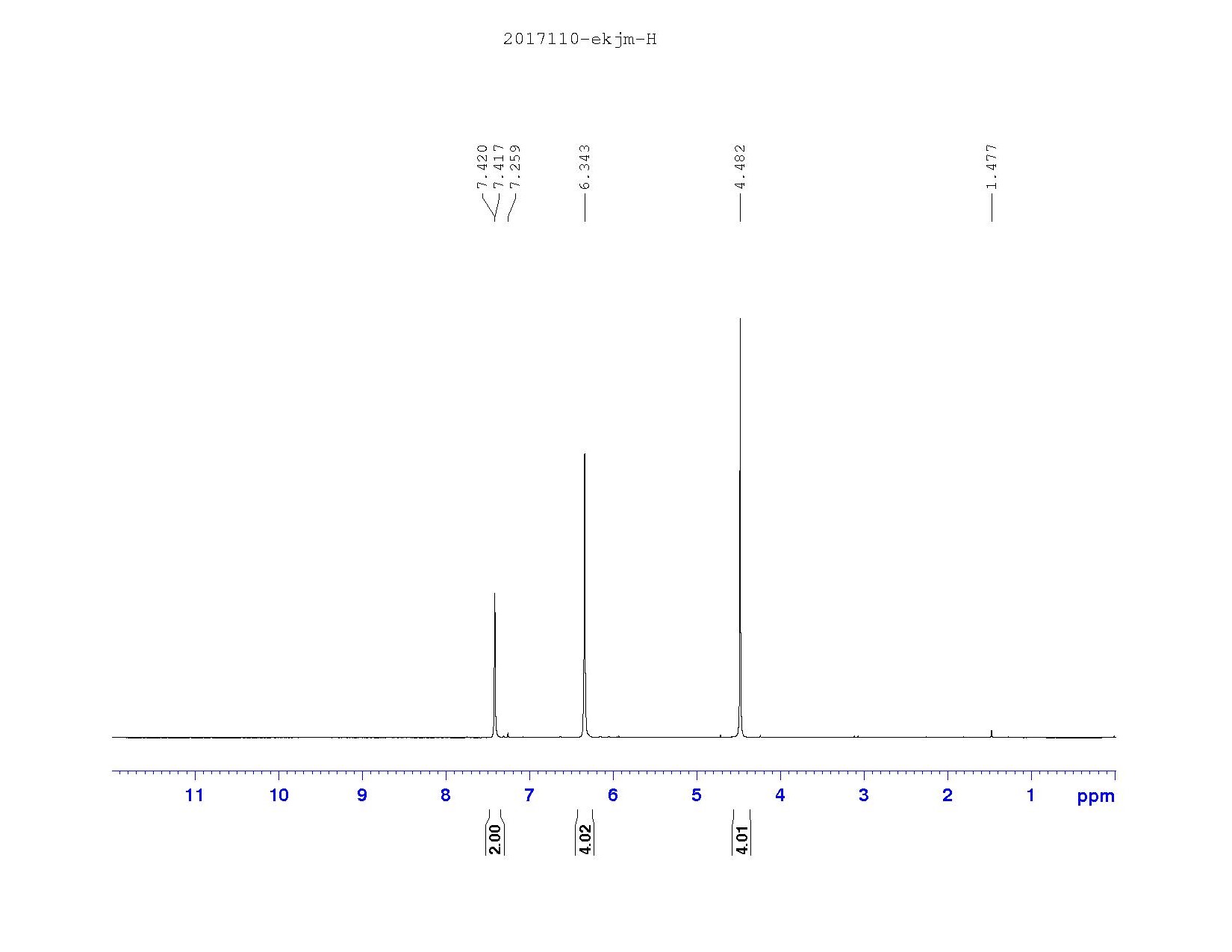


**S3. ^13^C NMR Spectra of 2, 2′-difurfuryl ether**


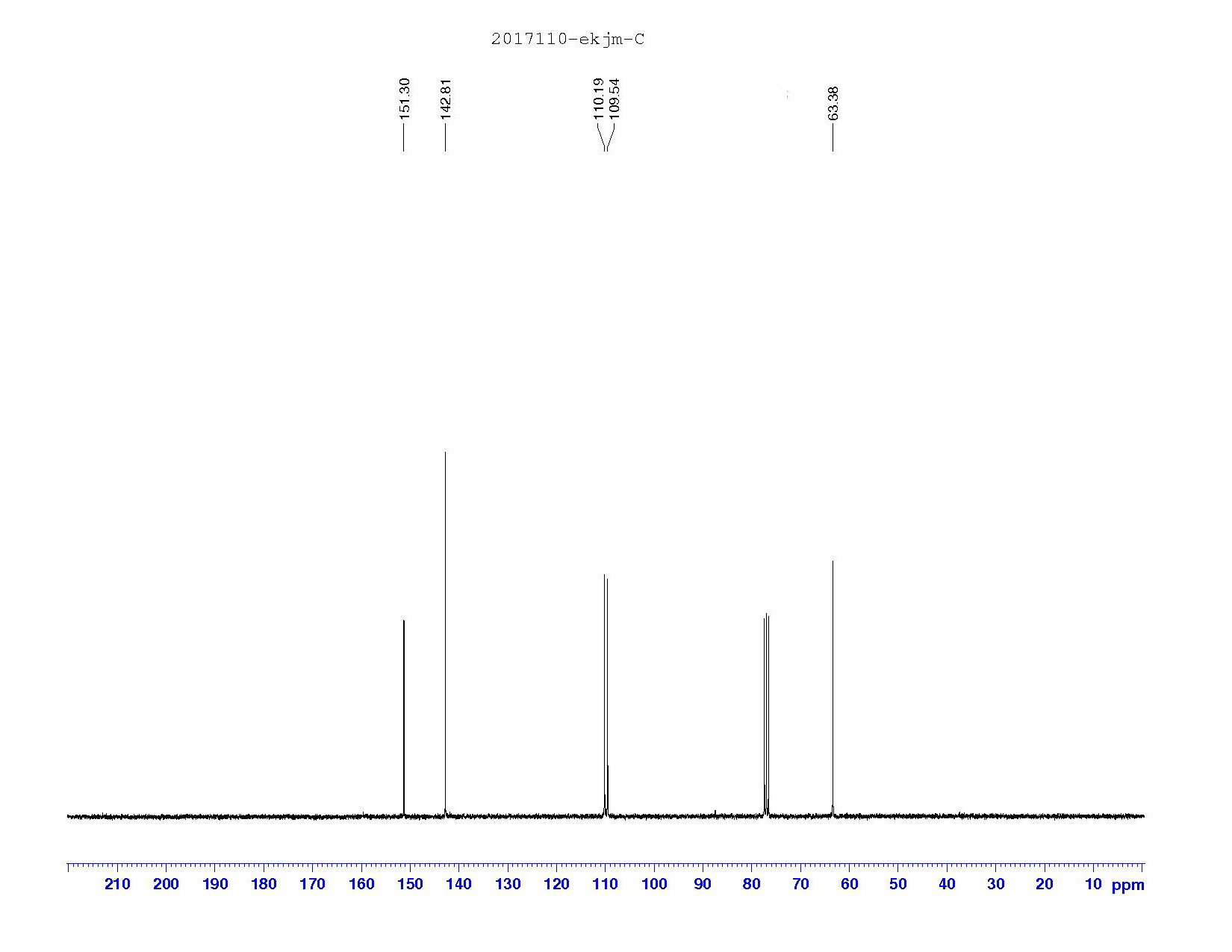


**S4. MS** **Spectra of 2, 2′-difurfuryl ether**

**S5. MS** **Spectra of compound 5**

**S6. MS** **Spectra of compound 6**

**S7. MS** **Spectra of compound 7**
